# Response to Statin Therapy in Obstructive Sleep Apnea Syndrome: A Multicenter Randomized Controlled Trial

**DOI:** 10.1155/2014/423120

**Published:** 2014-08-25

**Authors:** Marie Joyeux-Faure, Renaud Tamisier, Jean-Philippe Baguet, Sonia Dias-Domingos, Stephen Perrig, Georges Leftheriotis, Jean-Paul Janssens, Wojciech Trzepizur, Sandrine H. Launois, Françoise Stanke-Labesque, Patrick A. Lévy, Frédéric Gagnadoux, Jean-Louis Pepin

**Affiliations:** ^1^University Grenoble Alpes, HP2, Inserm U1042, Grenoble, France; ^2^CHU de Grenoble, Laboratoire EFCR, Clinique Universitaire de Physiologie, Grenoble, France; ^3^Laboratoire EFCR, Pôle Thorax et Vaisseaux, CHU de GRENOBLE CS 10217, 38043 Grenoble, France; ^4^CHU de Grenoble, Clinique de Cardiologie, Grenoble, France; ^5^Laboratoire du Sommeil, Service de Neuropsychiatrie, Hôpital Belle Idée, Genève, Switzerland; ^6^Laboratoire d'explorations Fonctionnelles Vasculaires, CHU d'Angers, Angers, France; ^7^Service de Pneumologie, Hôpital Universitaire de Genève, Genève, Switzerland; ^8^CHU d'Angers, Département de Pneumologie, Angers, France; ^9^Université d'Angers, Inserm 1063 “SOPAM”, Angers, France; ^10^CHU de Grenoble, Département de Biochimie, Toxicologie et Pharmacologie, Grenoble, France

## Abstract

*Rationale.* Accumulated evidence implicates sympathetic activation as inducing oxidative stress and systemic inflammation, which in turn lead to hypertension, endothelial dysfunction, and atherosclerosis in obstructive sleep apnea (OSA). Statins through their pleiotropic properties may modify inflammation, lipid profile, and cardiovascular outcomes in OSA. *Methods.* This multicenter, randomized, double-blind study compared the effects of atorvastatin 40 mg/day versus placebo over 12 weeks on endothelial function (the primary endpoint) measured by peripheral arterial tone (PAT). Secondary endpoints included office blood pressure (BP), early carotid atherosclerosis, arterial stiffness measured by pulse wave velocity (PWV), and metabolic parameters. *Results.* 51 severe OSA patients were randomized. Key demographics for the study population were age 54 ± 11 years, 21.6% female, and BMI 28.5 ± 4.5 kg/m^2^. In intention to treat analysis, mean PAT difference between atorvastatin and placebo groups was 0.008 (−0.29; 0.28), *P* = 0.979. Total and LDL cholesterol significantly improved with atorvastatin. Systolic BP significantly decreased with atorvastatin (mean difference: −6.34 mmHg (−12.68; −0.01), *P* = 0.050) whereas carotid atherosclerosis and PWV were unchanged compared to the placebo group. *Conclusion.* In OSA patients, 3 months of atorvastatin neither improved endothelial function nor reduced early signs of atherosclerosis although it lowered blood pressure and improved lipid profile. This trial is registered with NCT00669695.

## 1. Introduction

Obstructive sleep apnea (OSA) syndrome represents a serious health hazard and is recognized as an independent risk factor for adverse cardiovascular outcomes such as hypertension, arrhythmias, stroke, and coronary heart disease [1]. Sympathetic activation, oxidative stress, and systemic inflammation have been shown to be the main intermediary mechanisms linking intermittent hypoxia (IH), the marker of OSA, with deleterious cardiovascular and metabolic consequences leading to enhanced cardiovascular morbidity and mortality [2, 3]. Prior to the occurrence of cardiovascular events, sleep apnea is associated with several subclinical cardiovascular alterations including nocturnal hypertension and early atherosclerosis that are related to both vasculature remodeling (such as increased intima-media thickness [4], arterial plaque formation, and arterial stiffness [5–8]) and endothelial dysfunction [9]. OSA patients exhibit altered endothelial function with desensitization of the alpha and beta 2-adrenergic receptors, altered NO-dependent vasodilatation, and hypersensitivity to vasoconstriction (induced by angiotensin II (Ang II)) [10–12]. Alterations in endothelial function precede the development of morphological atherosclerotic changes and subsequent clinical complications [13]. Digital pulse amplitude augmentation in response to hyperemia (EndoPAT) is one of the validated methods for measuring endothelial function. EndoPAT has the advantage of being easy to perform compared to other endothelial function assessment techniques [14]. Measurements are automated with low interobserver and intraobserver variability. A main advantage of the system is that the contralateral arm serves as an internal control that can be used to correct for any systemic drift in vascular tone during the test. There is demonstration that peripheral arterial tone (PAT) values allow quantifying cardiovascular risk [15] and predicting late adverse cardiovascular events [16].

In OSA patients, reversing early disorders in the cardiovascular system before the occurrence of major clinical events, such as myocardial infarction or stroke, may be a means of reducing cardiovascular risk.

Continuous positive airway pressure (CPAP) the first line therapy for OSA has been suggested in small size randomized controlled trials (RCTs) as being able to reverse some of these subclinical alterations [17] as well as endothelial dysfunction [18]. However, CPAP acceptance is poor in some subgroups of OSA patients [19] and recent large RCTs demonstrate that CPAP alone is not enough to reduce cardiometabolic risk in OSA patients [20]. Thus a crucial issue is to develop alternative or combined treatments to address early, or delay, deleterious OSA-related cardiovascular consequences.

Statins were initially introduced for the prevention of cardiovascular risk because of their lowering lipid effects. During the last decade, numerous in vivo and in vitro studies have described pleiotropic effects of statins, independent of their lipid-lowering properties. Some of the reported pleiotropic effects of statins may impact intermediate mechanisms underlying cardiovascular risk in OSA patients. Simvastatin treatment is able to reduce sympathetic tone and normalize autonomic function in chronic heart failure (CHF) rabbits by inhibiting central Ang II mechanisms and therefore the superoxide pathway [21]. Statins are also able to reduce IH-induced hypertension to improve carotid compliance and to reduce cardiac infarction hypersensitivity on IH exposed rats [22]. These beneficial vascular effects have also been reported in normolipidemic patients with isolated systolic hypertension where statins reduce large artery stiffness and blood pressure [23]. Statins are also known to stabilize atherosclerosis plaques, induce inhibition of vascular smooth muscle cell proliferation as well as platelet aggregation, and reduce vascular inflammation [24–26].

Statins through their pleiotropic properties that impact intermediary mechanisms might modify cardiovascular outcomes in OSA. The aim of this study was thus to determine the effect of 3 months of atorvastatin treatment on endothelial function, blood pressure, and early signs of atherosclerosis in OSA patients, through a randomized double-blind placebo-controlled trial.

## 2. Material and Methods

The study was conducted in accordance with applicable good clinical practice requirements in Europe, French law, ICH E6 recommendations, and ethical principles of the Helsinki Declaration (South Africa 1996 and Edinburgh 2000). The study was approved by an independent ethics committee (Comité de Protection des Personnes, Grenoble, France, IRB0005578) and registered on the ClinicalTrials.gov site (NCT00669695). Written informed consent was obtained from all included patients. An external data quality control was performed systematically for some criteria (such as informed consent, complications, and adverse events) and by a random selection of 10% of the case report forms for other criteria.

### 2.1. Patients

This multicenter, randomized, double-blind, parallel group study compared atorvastatin 40 mg/day versus placebo over 12 weeks. The primary endpoint was change in endothelial function from baseline to 12 weeks, measured by PAT. Other endpoints included office blood pressure (BP), early carotid atherosclerosis (intima-media thickness (IMT) and carotid diameters), arterial stiffness measured by pulse wave velocity (PWV), and metabolic and inflammatory parameters.

Patients were recruited from sleep laboratories of 3 university hospitals (Grenoble and Angers, France and Geneva, Switzerland). Only subjects diagnosed with OSA (apnea-hypopnea index (AHI) > 30/h) aged over 18 years and who gave written informed consent were eligible. The study was conducted following the CONSORT recommendations [27].

Patients presenting any of the following criteria were not included: history of stroke, coronary heart disease, chronic respiratory failure, hypothyroidism, already on statin treatment, multiple antihypertensive medications, pregnant or lactating women, alcohol consumption > 3 units/day, treatment by itraconazole, ketoconazole, protease inhibitor, fibrates, antivitamin K, diltiazem, verapamil, erythromycin, clarithromycin, or cyclosporine.

The determination of the sample size was based on published data on the beneficial effect of an oral appliance using the same endpoint [28], showing that PAT improved from 1.77 ± 0.4 at baseline to 2.0 ± 0.4 after treatment. We hypothesized that the beneficial effect of statin treatment would be similar. A change of approximately 0.23 in this variable was anticipated after 3 months of atorvastatin treatment. No study to date has demonstrated the range of improvement in PAT values after an intervention that is predictive of reduced morbidity or mortality. The sample size calculated to obtain significant differences with 80% statistical power and an alpha error of 0.05 showed that 57 patients per group were necessary. Thus, 60 patients per group were initially planned. An interim analysis initially planned was scheduled after the inclusion of 25 patients per group.

### 2.2. Study Design and Treatment

#### 2.2.1. Baseline Visit

Patients underwent an overnight polysomnography. After waking up and while still in a fasting state, a peripheral blood sample was drawn. Then, endothelial function was assessed by PAT and arterial stiffness measured by PWV. A carotid ultrasonography was performed to assess IMT and carotid diameters. The Epworth sleepiness scale was completed and arterial blood gases analysis was performed in order to exclude obesity hypoventilation syndrome. Patients were then randomized to receive statin or placebo treatment. The randomization was made by an independent statistician. Investigators, patients, and the study team were blinded to treatment allocation. Patients randomly allocated to the statin group received 40 mg/day atorvastatin (Tahor, Pfizer Laboratories, France) during 12 weeks. Patients randomly allocated to the control group received placebo (lactose, LC2 Laboratories, France) similarly administered. In order to maintain the double blind status, atorvastatin tablets were encapsulated in capsules identical to the lactose placebo capsules (by LC2 Laboratories, France).

#### 2.2.2. Three-Month Visit

Twelve weeks after the baseline visit, the same parameters were measured to compare the effect of the statin treatment with placebo.

### 2.3. Study Outcomes

The primary endpoint was the effect of atorvastatin treatment on endothelial function between baseline and 12 weeks, as measured by PAT. The secondary objectives of the study were to determine the effect of atorvastatin treatment on BP, IMT and carotid diameters, arterial stiffness (PWV evaluation), and metabolic parameters. The primary analysis was in intention to treat.

### 2.4. Study Procedures

#### 2.4.1. Polysomnography (PSG)

Overnight sleep studies were scored manually according to standard criteria [29] and an AHI was calculated from the number of apneas and hypopneas per hour according to international guidelines [30].

#### 2.4.2. Blood Pressure Measurements

Clinical BP was measured using a mercury sphygmomanometer on three occasions, in line with European Society of Hypertension-European Society of Cardiology guidelines [31]. Systolic BP (SBP) and diastolic BP (DBP) were measured. Mean arterial BP (MABP) was calculated as DBP + 1/3(SBP-DBP).

#### 2.4.3. Endothelial Function

After BP measurements, endothelial function was assessed by reactive hyperaemia using the finger plethysmographic methodology (PAT) with the EndoPAT device (Itamar Medical Ltd, Caesarea, Israel) as previously described [32]. PAT index was calculated as the natural logarithm of the average amplitude of PAT signal 90 to 120 seconds after deflation divided by average amplitude of the PAT signal during the 210 seconds prior to cuff inflation [15].

*Carotid-to-femoral pulse wave velocity (PWV)* was used for the arterial stiffness evaluation. To determine the carotid-to-femoral PWV, two pulse transducers were fixed on the skin over the right common carotid and femoral arteries. The time delay was measured with a Complior device, between the troughs of simultaneously recorded pulse waves and averaged over 10 consecutive cycles. The carotid-femoral PWV was calculated as the distance between the arterial sites divided by the time delay. Increased PWV was defined as PWV > 12 m/s [33].

#### 2.4.4. Carotid Ultrasonography

B-mode ultrasonography was performed using an HP Sonos 2500 (Hewlett Packard) machine with a sectorial 7.5 MHz probe. The method used to determine the mean common carotid IMT and luminal diameter has been previously described [4]. Both common carotid arteries were studied consecutively in the long axis with a probe incidence allowing good quality images. The IMT was defined as the distance separating the most internal parts of these lines and the luminal diameter by the distance between the blood-intima interfaces on the anterior and posterior walls. The images were recorded in end-diastole and then analyzed by specific validated software (TIMC Laboratory, CHU Grenoble, France). IMT and diameter measurements were carried out on areas free of plaques and then averaged. The IMT and luminal diameter values were the mean values for the two common carotid arteries. Carotid ultrasonography was performed by two operators who were blinded to the other study data. The analysis of carotid parameters using the specific software was performed by the same operator throughout the entire study.

#### 2.4.5. Metabolic Parameters

After peripheral blood sampling, plasma glucose and serum triglyceride levels were measured automatically (Modular 700, Roche, Meylan, France). Serum insulin was measured using a radio-immunometric sandwich assay (CIS bio international, Gif-Sur-Yvette, France).

#### 2.4.6. Inflammatory Markers

The high-sensitivity C-reactive protein (hs-CRP) level was measured using automated immunonephelometry (Behring Nephelometer II Analyzer, Dade Behring, Germany). Urinary leukotriene E4 (LTE4, a validated marker of proinflammatory cysteinyl leukotriene production) and 11-dehydro-thromboxane B2 (11-DHTXB2) were quantified using liquid chromatography-tandem mass spectrometry [34].

### 2.5. Statistical Analysis

Statistical analysis was performed by using NCSS 97 software (Kaysville, Utah, USA). The data were analyzed in intention to treat, which includes all patients who signed the informed consent form. Missing baseline data were replaced by the median of each group and missing data at 12 weeks were replaced by the median of the opposite group (maximum bias method). Baseline data were compared by a Student or a Mann-Whitney test for continuous data (depending on the validity of the normality of distributions) and by a Chi-Square test for categorical data. For the analysis of data evolution between baseline and 12 weeks, a repeated measure two-way analysis of variance (ANOVA) was performed, followed by a Bonferroni post hoc test when necessary. When normality was not respected, a transformation of variables was used. All *P* values were two-tailed and a *P* value <0.05 was considered significant. Study design and data are reported here in accordance with the CONSORT criteria [27].

## 3. Results

### 3.1. Patient Characteristics

**Figure 1:**
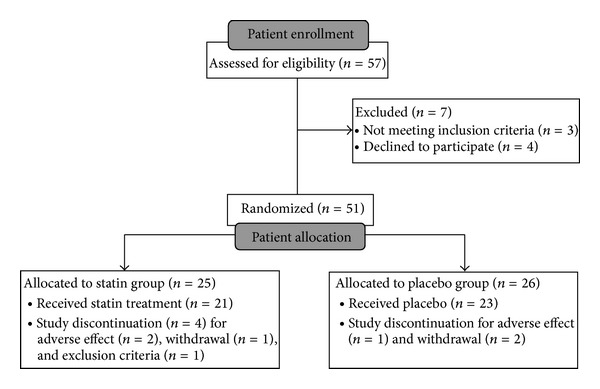


Fifty-one patients were included and randomized (*n* = 25 in the statin group, *n* = 26 in the placebo group) between 16 May 2008 and 1 June 2012 (36 patients in Grenoble between 16 May 2008 and 20 January 2012, 11 patients in Angers between 13 May 2011 and 1 June 2012, and 4 patients in Geneva between 16 February 2009 and 23 September 2010). Three patients (2 from the statin group and 1 from the placebo group) presented adverse effects such as myalgia or digestive disorders. Three patients withdrew: 1 from the statin group and 2 from the placebo group (see flow-chart presented in Figure 1).

Key demographics for the study population included age 54 ± 11 years, 21.6% female, BMI 28.5 ± 4.5 kg/m^2^. There were no significant difference regarding baseline demographic data between the groups and no differences in baseline BP and sleep apnea characteristics. None of the patients was hypercapnic. Baseline HDL cholesterol levels were significantly higher in the statin group (Table 1).

After 12 weeks of treatment, adherence was not significantly different between the two groups (94.7 ± 0.07% in the statin group versus 96.6 ± 0.05% in the placebo group).

### 3.2. Primary Outcome Analysis: Endothelial Dysfunction

After 12 weeks, there was no improvement in endothelial function when the statin intervention group was compared with the placebo group. The mean difference in PAT measurements between the groups was 0.008 (−0.29; 0.28), *P* = 0.979 (Table 2). This intermediary analysis, initially planned in the study protocol, revealed no positive effect of treatment on the primary endpoint. Moreover, as a simulated calculation with 3 times the number of included patients also showed no positive effect of treatment, the study was then stopped based on futility for primary outcome PAT measured endothelial function.

### 3.3. Secondary Outcome Analyses

#### 3.3.1. Change in Clinical BP

**Figure 2:**
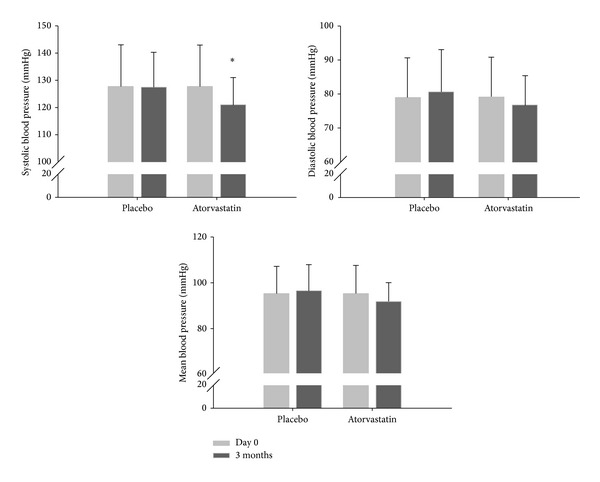


SBP significantly decreased after 12 weeks of atorvastatin treatment with a mean difference between groups of −6.34 mmHg (−12.68; −0.01), *P* = 0.050 (Table 3 and Figure 2).

#### 3.3.2. Effect on Arterial Stiffness

After 12 weeks, there was no effect of statin treatment in reducing arterial stiffness compared with the placebo group. The mean difference in PWV measurements between the groups was 0.54 m/s (−0.45; 1.52), *P* = 0.189 (Table 2).

#### 3.3.3. Effect on Carotid IMT and Diameters

After 12 weeks of treatment both carotid IMT and left and right luminal carotid diameters remained unchanged in both groups (Table 2).

#### 3.3.4. Changes in Biological Parameters

Total and LDL cholesterol levels significantly improved after 12 weeks of atorvastatin treatment (*P* < 0.0001), whereas HDL cholesterol was unchanged compared to the placebo group. Moreover, in both groups, glycemia, insulinemia, the HOMA index reflecting insulin resistance, and the glycated hemoglobin A1c level did not change (Table 4).

*Changes in Inflammatory Markers*. In both groups hs-CRP, LTE4, and 11-DHTXB2 were unchanged after 12 weeks of treatment (Table 5).

## 4. Discussion

This multicenter, randomized, double-blind, and parallel group study in OSA patients was the first to investigate the effect of statin treatment on OSA-related cardiovascular outcomes.

OSA impairs macro- and microvascular endothelial function compared to healthy controls. PAT reflects changes in digital microvessel dilatation which is only partly dependent on nitric oxide [14]. It has been demonstrated that PAT and flow-mediated dilatation (FMD) measurements have significant but differing relations with cardiovascular and metabolic risk factors. However, there is only a weak link between the two assessments [35]. In fact, FMD and PAT measure different aspects of vascular biology and provide distinct information regarding vascular function in conduit versus smaller digital vessels [35]. Using PAT, we showed that 3 months of atorvastatin neither improved endothelial function nor reduced early signs of atherosclerosis or arterial stiffness. In this relatively healthy population of OSA patients, we did not confirm the beneficial effect on vascular compliance that we have previously reported with statins in rats exposed to intermittent hypoxia [22]. This result is in accordance with another human study showing that, despite improvement in the lipid profile, 6 weeks of atorvastatin treatment (40 mg/day) failed to improve endothelial dysfunction in the first-degree relatives of patients with premature coronary artery disease [36]. The atorvastatin dosage used here (40 mg/day) may have potentially been too low to improve endothelial function and atherosclerosis markers in OSA patients. Indeed, a higher atorvastatin dosage (80 mg/day) was shown to improve endothelial function assessed by flow-mediated dilation [37] and to reduce large artery stiffness [23] in normolipidemic hypertensive patients. An alternative explanation might be that we did not include patients with comorbidities such that a majority of our OSA patients did not exhibit sufficient endothelial dysfunction or severe stiffening at baseline to detect an effect.

However and importantly, we showed that this statin dosage is able to lower systolic office blood pressure in OSA patients. This observation is in accordance with previous results from our group showing that in rodents statin treatment reduces IH-induced blood pressure elevation [22]. However, outside the area of sleep apnea, such a beneficial effect has also been reported in normolipidemic patients with isolated systolic hypertension [23, 37].

In vascular smooth muscle cells statins are known to inhibit hypoxia-induced endothelin-1 via accelerated degradation of HIF-1*α* [38]. Statins have also been shown, in the CHF rabbit, to reduce sympathetic tone, inhibiting central Ang II mechanisms and therefore the superoxide pathway [21]. There is growing evidence that NAD(P)H oxidase-derived reactive oxygen species induced by Ang II play an important role in the central regulation of autonomic activity and cardiovascular function in various pathological states. As previously shown in IH exposed rats [39], the hypertensive effect and cardiac infarction hypersensitivity induced by IH were abolished by antioxidant treatments (such as tempol and melatonin) which were able to normalize DHE level and NADPH expression. All these mechanisms might potentially be involved in the blood pressure lowering effect of statins observed here in OSA patients.

In this study, the impact of statin in reducing blood pressure (around 6 mmHg mean difference) is clinically relevant. This is particularly true in view of the limited impact of CPAP treatment in reducing BP [40, 41]. Indeed, among patients treated for hypertension, even 1 to 2 mmHg mean differences in office blood pressure are already associated with reduced odds of stroke and major cardiovascular events [42–44]. Law et al. have underlined the importance of lowering blood pressure in everyone over a certain age, rather than measuring it in everyone and treating it in some [44]. Larger reductions in blood pressure are known to produce larger reductions in the risk of all major cardiovascular events [43].

Finally, we also showed that statin treatment improved the lipid profile in normolipidemic OSA patients, in accordance with previous studies on normolipidemic hypertensive patients [23, 37]. We observed here that 3 months of statin treatment induced a decrease of 2 mmol/L in total cholesterol and of 1.68 mmol/L in LDL cholesterol that could significantly reduce cardiovascular risk. Indeed, lowering LDL cholesterol concentration by an average of 1.8 mmol/L under statins is able to reduce the risk of ischaemic heart disease events by about 60% and stroke by 17% [45].

Recent large RCTs demonstrate that CPAP alone is not sufficient to address cardiometabolic risk in OSA patients [20]. Thus, a combination of statin treatment with CPAP therapy could be useful to better control blood pressure in OSA patients and to reduce associated cardiovascular morbidity and mortality. Indeed, in view of the high risk of cardiovascular disease (CVD) in patients with OSA, the use of statins in this group of patients, irrespective of their baseline cholesterol levels, should be encouraged [46]. While absolute risk assessment is essential when considering primary prevention for CVD, an uncritical application of the Framingham risk equation may result in the underuse of statins in patients with OSA. Thus, when the Framingham risk tool is used to manage statin treatment for primary CVD prevention in OSA patients, a lower CVD risk threshold than that recommended by current guidelines may need to be set [46]. Evidence from large registries and long-term prospective trials is now required to determine the potential synergic beneficial effects and the rate of new cardiovascular events in patients with OSA receiving combined statin and CPAP therapy.

Finally, combined statin and CPAP therapy should be put in a realistic perspective compared to the undisputed effects of weight loss and/or exercise not only on blood pressure but also on the cardiometabolic consequences of sleep apnea [20, 47, 48].

## Figures and Tables

**Table 1:** Baseline characteristics of placebo and statin patients.

	Placebo	Statin	*P* value
*n*	26	25	
Age (years)	56 ± 9	51 ± 12	NS
Male gender (%)	73	84	NS
BMI (kg/m²)	28.70 ± 3.94	28.28 ± 5.12	NS
History			
Smoking (%)	50.0	60.0	NS
Alcohol (%)	38.5	56.0	NS
Diabetes (%)	0.0	4.0	NS
Dyslipidemia (%)	3.9	12.0	NS
Respiratory characteristics			
AHI (n/h)	45.47 ± 13.10	43.26 ± 19.19	NS
Mean SaO_2_ (%)	92.57 ± 1.44	92.70 ± 2.40	NS
SaO_2_ < 90% (%)	10.33 ± 13.20	11.66 ± 18.11	NS
Clinical BP			
SBP (mmHg)	127.67 ± 15.27	127.78 ± 15.07	NS
DBP (mmHg)	79.02 ± 11.61	79.16 ± 11.60	NS
MAP (mmHg)	95.22 ± 11.93	95.37 ± 12.21	NS
HR (bpm)	63 ± 8	64 ± 10	NS
Biological parameters			
Total cholesterol (g/L)	2.25 ± 0.64	2.46 ± 0.94	NS
LDL cholesterol (g/L)	1.41 ± 0.45	1.60 ± 0.67	NS
HDL cholesterol (g/L)	0.54 ± 0.23	0.74 ± 0.52∗	**0.011**
Triglycerides (g/L)	1.55 ± 1.10	1.16 ± 0.59	NS

Data are mean ± SD or percentage. BMI, body mass index; SaO_2_, oxygen saturation; SaO_2_ < 90%, percentage of recording time spent at a SaO_2_ < 90%; BP, blood pressure; SBP, systolic blood pressure; DBP, diastolic blood pressure; LDL, low-density lipoprotein; HDL, high-density lipoprotein; HR, heart rate. *P < 0.05 by Student or Mann-Whitney test. For qualitative data, a Chi-2 test or a Fisher test was performed. NS, not significant.

**Table 2:** Cardiovascular parameters at baseline (J0) and after 12 weeks (M3) of placebo or statin treatment.

	J0	M3	Change M3 − J0	Difference in change (95% CI)
PAT				
Placebo (*n* = 26)	2.09 ± 0.56	2.18 ± 0.45	0.09 ± 0.47	0.008 (−0.29; 0.28)
Statin (*n* = 25)	2.16 ± 0.62	2.24 ± 0.45	0.09 ± 0.54	
Carotid-to-femoral PWV (m/s)				
Placebo (*n* = 26)	9.41 ± 1.91	9.49 ± 1.88	0.08 ± 2.07	0.54 (−0.45; 1.52)
Statin (*n* = 25)	8.38 ± 1.33	8.99 ± 1.39	0.61 ± 1.37	
Right carotid IMT (*μ*m)				
Placebo (*n* = 26)	666.35 ± 165.33	667.96 ± 155.37	1.61 ± 155.91	0.02 (−70.38; 70.35)
Statin (*n* = 25)	615.00 ± 98.65	616.60 ± 92.99	1.60 ± 82.78	
Left carotid IMT (*μ*m)				
Placebo (*n* = 26)	726.54 ± 174.15	691.44 ± 130.30	−35.10 ± 123.69	17.42 (−45.98; 80.81)
Statin (*n* = 25)	676.68 ± 148.02	659.00 ± 112.14	−17.68 ± 99.81	
Mean carotid IMT (*μ*m)				
Placebo (*n* = 26)	696.73 ± 161.55	682.81 ± 130.51	−13.92 ± 119.44	5.34 (−51.34; 62.02)
Statin (*n* = 25)	647.16 ± 108.56	638.58 ± 93.26	−8.58 ± 77.68	
Right carotid luminal diameter (*μ*m)				
Placebo (*n* = 26)	6782 ± 1052	6539 ± 708	−243 ± 888	83 (−305; 472)
Statin (*n* = 25)	6515 ± 536	6355 ± 525^$^	−160 ± 396	
Left carotid luminal diameter (*μ*m)				
Placebo (*n* = 26)	6491 ± 816	6576 ± 770	85 ± 637	−73 (−360; 215)
Statin (*n* = 25)	6357 ± 527	6369 ± 602	12 ± 338	

Data are mean ± SD or percentage. PAT, peripheral arterial tone; PWV, pulse wave velocity; IMT, intima-media thickness. Analysis of data by repeated measure two-way ANOVA, followed by a Bonferroni post hoc test when necessary.

^$^ *P* < 0.05 for value visit.

**Table 3:** Blood pressure at baseline (J0) and after 12 weeks (M3) of placebo or statin treatment.

	J0	M3	Change M3 − J0	Difference in change (95% CI)
SBP (mmHg)				
Placebo (*n* = 26)	127.66 ± 15.26	127.46 ± 12.77	−0.21 ± 9.74	−6.34 (−12.68; −0.01)
Statin (*n* = 25)	127.78 ± 15.07	121.23 ± 9.68*	−6.55 ± 12.64	
DBP (mmHg)				
Placebo (*n* = 26)	79.02 ± 11.61	80.64 ± 12.33	1.62 ± 10.57	−3.98 (−9.98; 2.03)
Statin (*n* = 25)	79.16 ± 11.60	76.80 ± 8.55	−2.36 ± 10.78	
MAP (mmHg)				
Placebo (*n* = 26)	95.22 ± 11.93	96.31 ± 11.52	1.09 ± 9.17	−4.74 (−10.15; 0.67)
Statin (*n* = 25)	95.37 ± 12.21	91.72 ± 8.26	−3.65 ± 10.03	

Data are mean ± SD. SBP, systolic blood pressure; DBP, diastolic blood pressure; MAP, mean arterial pressure. *P < 0.05 versus baseline data by repeated measure two-way ANOVA, followed by a Bonferroni post hoc test when necessary.

**Table 4:** Metabolic parameters at baseline (J0) and after 12 weeks (M3) of placebo or statin treatment.

	J0	M3	Change M3 − J0	Difference in change (95% CI)
Total cholesterol (g/L)				
Placebo (*n* = 26)	2.25 ± 0.64	2.36 ± 1.24	0.11 ± 1.18	−0.89 (−1.50; −0.27)
Statin (*n* = 25)	2.45 ± 0.94^$^	1.68 ± 0.42^§^	−0.78 ± 0.99^£^	
LDL cholesterol (g/L)				
Placebo (*n* = 26)	1.41 ± 0.45	1.34 ± 0.46	−0.07 ± 0.38	−0.65 (−0.96; −0.34)
Statin (*n* = 25)	1.60 ± 0.66	0.88 ± 0.30	−0.72 ± 0.67	
HDL cholesterol (g/L)				
Placebo (*n* = 26)	0.54 ± 0.23	0.74 ± 0.91	0.21 ± 0.93	−0.31 (−0.78; 0.17)
Statin (*n* = 25)	0.74 ± 0.52	0.64 ± 0.49	−0.10 ± 0.74	
Triglycerides (g/L)				
Placebo (*n* = 26)	1.55 ± 1.10	1.27 ± 0.69	−0.28 ± 0.83	0.09 (−0.27; 0.46)
Statin (*n* = 25)	1.16 ± 0.59	0.97 ± 0.43	−0.18 ± 0.36	
Glycemia (mmol/L)				
Placebo (*n* = 26)	5.18 ± 0.51	5.03 ± 0.56	−0.15 ± 0.57	0.06 (−0.34; 0.46)
Statin (*n* = 25)	5.18 ± 0.76	5.01 ± 0.57	−0.09 ± 0.83	
Insulinemia (mUI/L)				
Placebo (*n* = 26)	9.84 ± 8.02	8.20 ± 6.15	−1.63 ± 5.12	−2.27 (−8.96; 4.42)
Statin (*n* = 25)	11.07 ± 16.38	7.16 ± 3.19	−3.90 ± 15.56	
HOMA				
Placebo (*n* = 26)	2.39 ± 2.05	1.83 ± 1.47	−0.56 ± 1.42	−0.66 (−2.83; 1.51)
Statin (*n* = 25)	2.83 ± 5.19	1.61 ± 0.77	−1.22 ± 5.11	
Hemoglobin A1c (%)				
Placebo (*n* = 26)	5.60 ± 0.42	5.64 ± 0.40	0.04 ± 0.25	0.04 (−0.12; 0.19)
Statin (*n* = 25)	5.64 ± 0.41	5.71 ± 0.43	0.08 ± 0.30	

Data are mean ± SD. LDL, low-density lipoprotein; HDL, high-density lipoprotein; HOMA, homeostasis model assessment of insulin resistance.

^$^ *P* < 0.05 for value group, ^§^ *P* < 0.0001 value visit, ^£^ *P* < 0.0001 value interaction, by repeated measure two-way ANOVA, followed by a Bonferroni post hoc test when necessary.

**Table 5:** Inflammatory parameters at baseline (J0) and after 12 weeks (M3) of placebo or statin treatment.

	J0	M3	Change M3 − J0	Difference in change (95% CI)
hs-CRP (mg/L)				
Placebo (*n* = 26)	3.9 ± 4.0	2.4 ± 1.8	−1.5 ± 4.4	2.5 (−3.8; 8.8)
Statin (*n* = 25)	3.8 ± 6.1	4.8 ± 13.7	1.0 ± 15.4	
Urinary LTE4 (pg/mg creatinine)				
Placebo (*n* = 26)	83.8 ± 72.9	88.4 ± 73.7	4.6 ± 42.8	14.1 (−10.4; 38.6)
Statin (*n* = 25)	68.8 ± 31.1	87.5 ± 50.9	18.7 ± 44.4	
Urinary 11-DHTXB2 (pg/mg creatinine)				
Placebo (*n* = 26)	784.5 ± 463.5	848.0 ± 635.9	63.6 ± 355.6	41.0 (−156.9; 238.9)
Statin (*n* = 25)	704.6 ± 279.9	809.1 ± 374.6	104.6 ± 347.2	

Data are mean ± SD. hs-CRP, high-sensitivity C-reactive protein; LTE4, leukotriene E4; 11-DHTXB2, 11-dehydro- thromboxane B2; TXB2, thromboxane B2.
